# Delirium in critically ill COVID-19 patients: incidence, predictors, and outcomes in invasive mechanical ventilation with versus without ECMO support

**DOI:** 10.1186/s13019-026-04057-1

**Published:** 2026-04-15

**Authors:** Stella Seeger, Ismail Dalyanoglu, Johannes Nienhaus, Esma Yilmaz, Luis Jaime Vallejo Castano, Anna Maria Markser, Bernhard Korbmacher, Artur Lichtenberg, Hannan Dalyanoglu

**Affiliations:** 1 Department of Cardiac Surgery, Medical Faculty, University Hospital Duesseldorf, Heinrich-Heine-University Duesseldorf, Duesseldorf, Germany; 2 Department of Vascular- and Endovascular Surgery, Marienhospital Gelsenkirchen, Gelsenkirchen, Germany; 3 Department of Anaesthesiology, Medical Faculty, University Hospital Duesseldorf, Heinrich-Heine-University Duesseldorf, Moorenstr. 5, 40225 Duesseldorf, Germany; 4 Clinical Institute for Psychosomatic Medicine and Psychotherapy, Medical Faculty, University Hospital Duesseldorf, Heinrich-Heine-University, Duesseldorf, Germany

**Keywords:** COVID-19, Delirium, ECMO, Mechanical ventilation, Sedation, Cardiothoracic ICU, Long-term outcomes

## Abstract

**Background:**

Delirium is a frequent and serious complication in critically ill COVID-19 patients, especially those requiring prolonged mechanical ventilation (IMV) or extracorporeal membrane oxygenation (ECMO). Given its negative impact on morbidity and mortality in the ICU, understanding the prevalence, risk factors, and long-term effects of delirium in cardiothoracic ICU patients remains crucial. This study aimed to determine delirium prevalence, identify independent predictors, and assess its association with short- and long-term outcomes in a cohort with a high proportion of prolonged weaning and ECMO support.

**Materials and methods:**

In this single-center cohort study, adult COVID-19 patients admitted between March 2020 and December 2021 requiring ≥ 72 h of invasive mechanical ventilation (IMV) and/or ECMO were included. Delirium was assessed retrospectively using daily CAM-ICU scores, Richmond Agitation-Sedation Scale (RASS) documentation, and structured chart review to capture hypoactive and hyperactive forms. Clinical data, sedation, analgesia exposure, and ICU management parameters were collected. Multivariable logistic regression identified predictors; a second model included ICU length of stay to account for potential bidirectional associations. Time-dependent Cox regression was used in mortality analyses to reduce survivorship bias. Long-term psychological outcomes were evaluated 6–12 months post-discharge.

**Results:**

Among 102 patients (58 ECMO, 44 IMV only), 53.9% developed delirium. Prevalence was higher in the IMV-only group (63.6%) than in the IMV–ECMO group (46.6%; *p* = 0.065). Multivariable analysis showed that lack of ECMO support, male sex, and longer duration of invasive ventilation were associated with increased delirium risk. When ICU length of stay was included, only absence of ECMO and ICU length of stay remained significant, while male sex lost significance. Thirty-day survival was higher in delirious patients (*p* = 0.039), but this was not confirmed for overall survival or in time-dependent analyses. No statistically significant differences were found in long-term psychological outcomes, although no statistically significant differences were observed between patients with and without delirium.

**Conclusion:**

Delirium is common among prolonged IMV and ECMO COVID-19 patients in cardiothoracic ICUs. Differences in incidence between ECMO and IMV-only patients may reflect differences in care protocols rather than illness severity alone, but no causal conclusions can be drawn from our data. Therefore, we suggest modifying potentially preventative factors such as sedation practices, and emphasize the need for future prospective studies.

## Introduction

Delirium is a frequent and severe acute brain dysfunction in critically ill patients, especially following cardiac surgery during postoperative intensive care. It manifests as fluctuating disturbances in attention, awareness, and cognition, and is increasingly recognized as an independent predictor of poor outcomes. Pathophysiological mechanisms include neuroinflammation, neurotransmitter imbalance, oxidative stress, and cerebral microvascular injury, all of which may be amplified in severe infections such as Coronavirus Disease 2019 (COVID-19) [1].

During the COVID-19 pandemic, patients with advanced cardiovascular disease or undergoing cardiac surgery faced disproportionately high risks of severe respiratory failure, often requiring prolonged invasive mechanical ventilation (IMV) or extracorporeal membrane oxygenation (ECMO) support for cardiac or respiratory failure. Both interventions are linked to an elevated delirium risk due to deep or prolonged sedation, systemic inflammation, and critical illness–related factors [2–4].

Multiple studies have demonstrated that sedative choice—particularly benzodiazepine exposure—sedation depth, hypoxemia, and systemic inflammation are strong, modifiable risk factors for delirium [5–7]. Preventive strategies such as the ABCDEF bundle effectively reduce delirium incidence and duration in Intensive Care Unit (ICU) patients. However, specific data on delirium in cardiothoracic ICU patient populations, especially with severe COVID-19, remain limited.

ECMO patients represent a distinct subgroup. Although typically representing patients with more severe respiratory and/or cardiopulmonary failure compared with invasively ventilated patients not requiring ECMO, their sedation may be based on different protocols, including the including the infrequently used awake ECMO strategy (i.e., ECMO support with minimized or no continuous deep sedation), which allows for lighter sedation, early mobilization, and may potentially reduce delirium incidence [8]. In cardiothoracic ICUs, additional factors—such as cardiopulmonary bypass–related cerebral microembolization, postoperative inflammatory response, and pre-existing cardiovascular comorbidities—may further increase delirium risk [2, 9]. Despite the high clinical relevance, there is limited evidence on the epidemiology, predictors, and long-term consequences of delirium in COVID-19 patients treated in cardiothoracic ICUs, especially those requiring prolonged IMV or ECMO. Understanding delirium in this cohort is crucial because:

1. It is associated with worse long-term neurocognitive and psychological outcomes, including depression and post-traumatic stress disorder [6].
2. Modifiable risk factors such as sedation depth and sedative class can be mitigated through protocolized care.
3. Differences in sedation and mobilization practices between IMV-only and ECMO patients may influence delirium incidence independently of illness severity [6, 10].

### Aim of this study

(1) To determine the prevalence of delirium, (2) identify independent predictors with a focus on modifiable factors including sedation practices, and (3) assess its association with short-term mortality and long-term psychological outcomes in COVID-19 patients requiring prolonged IMV with or without ECMO in a cardiothoracic ICU. We hypothesized that ECMO patients may have lower delirium incidence despite higher baseline illness severity supported by distinct sedation and mobilization protocols.

## Methods

### Study design and patient population

This retrospective observational single-center study included adult patients (≥ 18 years) with laboratory-confirmed SARS-CoV-2 infection admitted to the cardiothoracic ICU between March 2020 and December 2021. Importantly, none of the included patients were postoperative cardiac‑surgery patients. All admissions represented primary, critically ill COVID‑19 cases requiring advanced respiratory or cardiopulmonary support. Transfers to the cardiothoracic ICU occurred due to severity and the unit’s ECMO expertise, not due to postoperative care or general ICU capacity reasons. Because the cohort consisted exclusively of primary COVID‑19 ICU patients and not postoperative cardiac‑surgery cases, surgical variables such as procedure type, surgical indication, urgency, or postoperative complications were not applicable and therefore not collected. Patients were eligible if they required invasive mechanical ventilation (IMV) for ≥ 72 h and/or ECMO support. ECMO modality was classified as either veno‑venous (V‑V) ECMO for isolated respiratory failure or veno‑arterial (V‑A) ECMO when circulatory support was required. Both modalities were available within the cardiothoracic ICU. For the purpose of this study, IMV lasting ≥ 72 h was defined as prolonged IMV Mechanical ventilation in this study refers exclusively to invasive mechanical ventilation (IMV); non-invasive ventilation was not included. For all analyses, patients were categorized into two predefined groups:

(1) IMV–ECMO, comprising patients who received extracorporeal membrane oxygenation (ECMO) support during their ICU stay in addition to invasive mechanical ventilation (IMV); and (2) IMV-only, comprising patients treated with IMV without ECMO support.

All patients receiving ECMO support in this cohort required invasive mechanical ventilation during their ICU course; no patient received ECMO without IMV These group definitions were applied consistently throughout the manuscript. Delirium was diagnosed retrospectively via medical records review, using available Confusion Assessment Method for ICU (CAM-ICU) and Richmond Agitation Sedation Score (RASS) data. Exclusion criteria were pre-existing severe cognitive impairment, documented coma persisting until death, or missing key outcome data.

### Delirium assessment

Delirium was retrospectively assessed based on systemic chart review, guided by CAM-ICU criteria in clinical documentation. Clinical and demographic variables including age, sex, comorbidities (e.g., Chronic Obstructive Pulmonary Disease (COPD), ICU interventions, and length of stay were recorded.

Prospective follow-up involved validated instruments for assessment of psychological parameters: BDI-II, ITQ, SF-12, and ECR-R8:

- The Beck Depression Inventory-II (BDI-II): A 21-item self-report scale with scores ranging from 0 to 63. It has excellent internal consistency (Cronbach’s α ≈ 0.91) and test-retest reliability (*r* = 0.93), correlating strongly with other depression scales such as the BDI-IA (*r* = 0.93) and the Hamilton Depression Rating Scale (*r* = 0.71) [11].
- The International Trauma Questionnaire (ITQ): An 18-item measure assessing post traumatic stress disorders (PTSD) and complex PTSD symptoms per International Classification of Disease − 11 (ICD-11) criteria. The ITQ shows high internal consistency (α = 0.89–0.94) and diagnostic performance comparable to the PCL-5 [12].
- The SF-12 Health Survey: Evaluates physical (PCS) and mental (MCS) health-related quality of life, correlating strongly with the SF-36 (PCS: *r* = 0.951; MCS: *r* = 0.969) and demonstrating good internal consistency (PCS: α = 0.76; MCS: α = 0.89) [13].
- The ECR-R8 is an 8-item version of the ECR-RD used to assess attachment-related anxiety and avoidance. It correlates highly with the original version (anxiety: *r* = 0.92; avoidance: *r* = 0.90) and demonstrates good validity. Short forms with comparable content report internal consistency values around α = 0.87–0.88 [14, 15].

### Sedation and analgesia data

Given the known impact of sedative choice and depth on delirium risk, daily exposure data were extracted for:

- Benzodiazepines (Midazolam, Lorazepam; cumulative dose).
- Propofol (cumulative dose).
- Dexmedetomidine (cumulative dose).
- Opioids (Fentanyl, Sufentanil, Remifentanil; cumulative dose).
- Neuromuscular blocking agents (NMBA) – use (yes/no, duration).
- Sedation depth (summary of the median daily RASS score).

### Other clinical variables

Demographic data (age, sex), comorbidities (Charlson Comorbidity Index), illness severity (SOFA score, PaO₂/FiO₂ ratio at admission), and ICU interventions (prone positioning, ECMO, renal replacement therapy) were recorded. The primary indication for invasive mechanical ventilation and ECMO support in this cohort was COVID‑19‑associated hypoxemic respiratory failure. Veno‑venous (V‑V) ECMO was applied in cases of isolated respiratory failure, whereas veno‑arterial (V‑A) ECMO was used only when cardiogenic shock or combined respiratory–circulatory failure was present.

### Outcomes

- Primary outcome: Incidence of delirium during ICU stay.
- Secondary outcomes: 30-day and in-hospital mortality, duration of mechanical ventilation, ICU and hospital length of stay, and long-term psychological outcomes (depression, PTSD, quality of life, attachment style) assessed post-discharge using BDI-II, ITQ, SF-12, and ECR-R8.

Mortality was assessed using existing time‑to‑event analyses (including a time‑dependent Cox approach), and no additional derived parameters such as ventilator‑free or ECMO‑free days were available due to the retrospective dataset structure. Treatment‑modality‑specific mortality (IMV‑only vs. IMV–ECMO) could therefore only be addressed descriptively within the existing statistical framework. Group‑level mortality differences between IMV‑only and IMV–ECMO patients could not be analyzed using ventilator‑free or ECMO‑free day metrics due to unavailable data; mortality was therefore evaluated within the existing survival analyses already presented in the main result.

### Follow-up procedures

Survivors were contacted via mail and/or telephone 6–12 months after hospital discharge. The follow-up rate was calculated as the number of respondents divided by eligible survivors. Missing questionnaire data were handled using multiple imputation when > 5% of data points were missing for a given scale.

### Ethics

The study was approved by the local ethics committee (Heinrich Heine University Düsseldorf, Protocol #2023–2566, December 6, 2023) and conducted in accordance with the Declaration of Helsinki. Due to the retrospective and anonymized design, patient consent was waived.

### Statistical analysis

Statistical analyses were performed using IBM SPSS Statistics (Version 29.0.2.0). Kaplan-Meier analyses were created using R (Version R 4.5.1 GUI 1.82). Continuous variables are presented as median, interquartile range (IQR) or mean ± standard deviation (SD) as appropriate, categorical variables as n (%). Group comparisons were made using Mann–Whitney U or Student’s t-test for continuous variables and χ² or Fisher’s exact test for categorical variables. Multivariable logistic regression was used to identify independent predictors of delirium, including age, sex, Sequential Organ Failure Assesment score (SOFA), ventilation duration, ECMO status, sedative/analgesic exposure, and NMBA use. For mortality analysis, a time-dependent Cox proportional hazards regression was applied with delirium as a time-varying covariate to account for survivorship bias. Collinearity was assessed using variance inflation factor (VIF), with variables with VIF > 5 excluded.

## Results

A total of 102 patients were included (58 ECMO, 44 mechanical ventilation only). Delirium was diagnosed in 53.9% of patients, with all 102 included patients being evaluable for delirium assessment. Prevalence was higher in the IMV-only ECMO group (63.6% vs. 46.6%, *p* = 0.065). The “delirium present” group comprised 43 male subjects, while the “delirium absent” group included 29. Patients with delirium were older than those without delirium (mean age 59.38 vs. 55.68 years), although this difference did not reach statistical significance (*p* = 0.136). The increased incidence of coronary heart disease observed in the delirium group did not reach statistical significance (*p* = 0.060). However, a significant association was identified between ICU length of stay and the occurrence of delirium (*p* = 0.022).

The absence of ECMO support (*p* = 0.020), male sex (*p* = 0.008), and longer duration of invasive mechanical ventilation (*p* = 0.036) were identified as significant independent predictors of delirium. In a second regression model including ICU length of stay, the absence of ECMO support remained a significant predictor of delirium (*p* = 0.020), whereas male sex was no longer significant; ICU length of stay emerged as a significant predictor (*p* = 0.022). Conversely, male sex was no longer a significant predictor (*p* > 0.05), while ICU length of stay emerged as a significant predictor of delirium (*p* = 0.022). In consideration of the potential bidirectional relationship between delirium and length of stay in intensive care, both models were reported.

Thirty-day survival was significantly higher in the delirium group compared with the non-delirium group (*p* = 0.039); however, no significant difference was observed in overall survival (*p* = 0.400).

No significant group differences were identified in the psychosomatic parameters under investigation. Patients with delirium had lower mean BDI-II scores (10.15 vs. 15.45), lower SF-12 PCS scores (39.89 vs. 44.03), higher SF-12 MCS scores (45.39 vs. 42.90), and higher ECR-R8 anxiety T-scores (51.69 vs. 49.54); however, none of these differences reached statistical significance (all *p* > 0.05).

## Discussion

### Incidence of delirium in critically ill COVID-19 patients

Our findings confirm a high incidence of delirium in critically ill COVID-19 patients, with an overall prevalence of 53.9%, consistent with prior reports in severe COVID-19 cohorts [2, 5, 7]. Notably, ECMO patients in our study exhibited a non-significant trend toward lower delirium rates (46.6% vs. 63.6%, *p* = 0.065) despite higher illness severity at baseline.

### Predictors of delirium and role of ECMO support

While this observation aligns with reports suggesting that lighter sedation and early mobilization utilizing different *awake ECMO* protocols may reduce delirium risk [8], our study did not collect detailed, protocol-specific sedation or mobilization data for either of the observed groups. The proportion of patients receiving ECMO in this cohort is higher than in population‑based COVID‑19 studies because the cardiothoracic ICU functions as a regional referral center for severe respiratory failure. Consequently, the case‑mix is enriched toward the most critically ill COVID‑19 patients, many of whom were transferred for ECMO evaluation, leading to a higher ECMO utilization rate than in general ICU cohorts. Therefore, any explanation based on differences in care protocols remains speculative and should be interpreted cautiously. Nevertheless, recent multicenter evidence provides indirect support for the concept that ECMO management strategies may influence neurocognitive outcomes. A systematic review and meta-analysis of awake ECMO in cardiogenic shock demonstrated a significant reduction in mortality (OR = 0.28; 95% CI: 0.15–0.49) without an increase in secondary complications such as ventilator-associated pneumonia or infection rates, suggesting that lighter sedation and preserved mobility can be safely achieved in selected ECMO populations, although all included studies were retrospective [1, 5, 7, 16–18]. In this context, a Korean review describing awake ECMO (ECMO support with preserved patient interaction and spontaneous breathing) as a bridge to lung transplantation highlighted practical advantages including reduced sedative exposure and maintenance of patient mobility, both of which are established protective factors against delirium [19]. Taken together, these observations support the plausibility that ECMO strategies emphasizing lighter sedation and mobilization may modulate delirium risk, while underscoring the need for prospective studies specifically addressing neurocognitive outcomes.

### Pharmacokinetic considerations during ECMO and implications for delirium

Extracorporeal membrane oxygenation is known to substantially alter the pharmacokinetics of sedative and analgesic agents. Drug sequestration within the ECMO circuit—particularly within the oxygenator membrane and tubing—has been demonstrated for lipophilic and highly protein-bound substances, including benzodiazepines, opioids, and propofol. This phenomenon may result in reduced bioavailability, unpredictable plasma concentrations, and delayed onset or offset of drug effects compared with non-ECMO patients, often necessitating higher cumulative doses to achieve target sedation levels.

In the context of delirium, these pharmacokinetic alterations are clinically relevant, as increased cumulative exposure to sedatives—especially benzodiazepines—is a well-established risk factor for ICU delirium. Conversely, strategies aiming to minimize intravenous sedative exposure during ECMO, including lighter sedation targets and the use of alternative agents such as dexmedetomidine, may mitigate delirium risk.

Furthermore, in selected patients managed with awake ECMO concepts, the use of non-intravenous routes of drug administration (e.g., enteral analgesia or anxiolysis) may partially circumvent circuit-related drug sequestration and contribute to more stable sedation profiles. Compared with IMV-only patients, ECMO-supported patients are therefore exposed to a unique interplay of altered pharmacokinetics, sedation strategies, and mobilization practices, which may influence delirium incidence independently of illness severity.

### Comparison with non-COVID cardiac surgery cohorts

Our results can be compared to cardiac surgery patients without COVID-19. In cardiac surgery patients without COVID-19, reported delirium incidence ranges from 10% to 50%, depending on surgical technique, patient comorbidities, and delirium assessment criteria [10]. The higher delirium incidence observed in our cohort may be related to COVID-19–associated systemic inflammation, longer ICU stays, and increased cumulative exposure to sedative agents. In this context, the role of optimized sedation protocols in delirium prevention is critical. The PADIS Guidelines (Pain, Agitation/Sedation, Delirium, Immobility, Sleep Disruption) of the Society of Critical Care Medicine recommend strategies such as targeting light sedation, avoiding benzodiazepines when possible due to their association with increased delirium risk, using dexmedetomidine in agitated delirium because of its lower respiratory depression potential and favorable delirium profile, performing daily sedation interruptions (Spontaneous Awakening Trials), and integrating non-pharmacological measures including patient reorientation, sleep hygiene protocols, and early mobilization [20]. These measures have the potential to reduce delirium incidence and duration and are reasonable to be considered in any ICU population at risk, including COVID-19 patients.

### Clinical and outcome relevance of delirium

Importantly, large multicenter efforts such as the DECAF (Delirium after cardiac surgery) observational studies corroborate the association of delirium with prolonged ICU length of stay, increased mortality, and higher rates of discharge to care facilities [21, 22]. These parallels underscore that delirium in COVID-19 cardiothoracic ICU patients extends beyond a neurological complication, substantially affecting resource utilization and clinical outcomes.

### Delirium motor subtypes and diagnostic challenges

A critical aspect often insufficiently addressed in ICU delirium research is the heterogeneity of delirium motor subtypes, which include hypoactive, hyperactive, and mixed forms. Hypoactive delirium, characterized by reduced motor activity, lethargy, and diminished responsiveness, frequently goes underdiagnosed due to its subtle clinical presentation but is associated with poor outcomes equal to or worse than hyperactive delirium [23]. Given the retrospective nature of our assessment, relying mainly on chart review and CAM-ICU documentation, underrecognition of hypoactive delirium is a plausible limitation. Differentiating delirium subtypes prospectively would enhance risk stratification and inform precise therapeutic approaches, since sedation regimens and mobilization strategies may differentially impact the prevalence and severity of delirium subtypes.

### Long-term psychological and quality-of-life outcomes

Extensive literature documents that delirium during critical illness – including COVID-19 - can lead to persistent neurocognitive impairments lasting months to years post-discharge [6]. Critical illness and intensive care unit (ICU) admission as well as severe COVID-19 have been linked to persistent psychological symptoms, such as PTSD, depression, and diminished quality of life [9, 24]. Moreover, existing literature indicates that delirium is significantly associated with psychological distress at follow-up [25], and preliminary findings suggest that such distress may be linked to poorer self-reported cognitive functioning after delirium [25].

The absence of significant differences in psychometric assessments between the delirium and non-delirium groups in our sample likely reflects limitations in sample size and follow-up duration rather than true equivalency. This highlights the urgent need for rigorous, long-term prospective neuropsychological follow-up to capture both subtle cognitive deficits and psychological symptoms, thereby informing appropriate rehabilitation strategies. For future research, continuous post-ICU monitoring of cognitive and psychological outcomes remains essential to fully appreciate and mitigate the long-term burden of delirium in this vulnerable population [6].

### Clinical implications and future directions

Our results highlight the pivotal role of modifiable sedation factors, including depth and agent selection, in delirium pathogenesis, thereby opening promising avenues for intervention-based research. Optimizing sedation protocols adapted to COVID-19 patients requiring ECMO or prolonged IMV—coupled with early mobilization and awake ECMO strategies—has demonstrated feasibility and potential efficacy in reducing delirium as part of comprehensive care bundles [1, 8]. Future randomized controlled trials and multicenter prospective studies are imperative to clarify the mechanisms, confirm benefits, and establish evidence-based ICU guidelines integrating sedation management and delirium prevention within complex cardiothoracic and COVID-19 critical care settings.

### Delirium subtypes, sedation complexity, and prognostic implications

The integration of delirium motor subtype differentiation remains essential to improving clinical recognition and management. The often silent presentation of hypoactive delirium complicates diagnosis but carries significant mortality and morbidity risks, while hyperactive and mixed delirium forms may prompt more immediate intervention due to their overt symptoms. The complexity of pharmacological management is reflected in varied interventions across subtypes, with evidence suggesting that inappropriate sedation may worsen outcomes by inducing transitions between subtypes, prolonging ICU stay, and increasing mortality. These insights highlight the necessity for nuanced approaches to sedation and delirium treatment to improve prognoses.

### Health system and resource implications

From a systems perspective, delirium has substantial economic and resource implications. It is associated with longer IMV duration, extended ICU and hospital stays, and increased post-acute care utilization, all of which increase direct healthcare costs and burden ICU capacity [26, 27]. This was particularly relevant during the COVID-19 pandemic, when ICU resources—especially in specialized cardiothoracic units with ECMO capability—were strained. Such measures offer dual benefits of improving patient outcomes and optimizing ICU resource allocation across healthcare systems [26, 27].

Furthermore, the focus on COVID-19 patients within a cardiothoracic ICU limits generalizability to broader ICU populations. These considerations reinforce the need for prospective, standardized delirium assessment protocols tailored to specialized ICU cohorts.

### Clinical implications and future directions

Our findings underscore the critical importance of routine delirium detection in cardiac intensive care units, particularly for survivors of ECMO and patients undergoing prolonged mechanical ventilation. The high prevalence of delirium in this specific cohort necessitates standardized implementation of validated delirium screening tools such as the Confusion Assessment Method for the ICU (CAM-ICU) as part of usual care. Early and reliable identification of delirium enables prompt therapeutic interventions and may mitigate adverse outcomes. In addition to systematic screening, protocols aimed at minimizing sedation depth, encouraging early mobilization, and fostering active family engagement should be employed to reduce delirium incidence and severity. These multidimensional strategies are supported by emerging evidence highlighting modifiable risk factors that can be targeted during critical illness [1, 8]. Moreover, structured post-ICU care pathways incorporating psychological evaluation and support are essential, given the potential for persistent neurocognitive and psychological sequelae following ICU delirium, as suggested by current literature and our study findings.

Further research should explore the complex interplay between surgical complexity, inflammatory response, sedation strategies, and delirium risk in cardiothoracic ICU patients. Large multicenter prospective studies that differentiate delirium motor subtypes could refine risk stratification and enable precision prevention. Interventional trials on sedation optimization, *awake ECMO* protocols, and early rehabilitation are urgently needed to establish evidence-based guidelines tailored to COVID-19 and cardiothoracic ICU populations.

### Limitations

This study’s retrospective and single-center design inherently limits the external validity and interpretability of the results. Delirium assessment relied on documented CAM-ICU and Richmond Agitation-Sedation Scale (RASS) scores as well as chart review, which carries the risk of underestimating true delirium incidence, especially of the hypoactive subtype that often remains clinically subtle. Variability in clinical documentation and potential charting bias further contribute to this challenge. The relatively small sample size may have constrained statistical power to detect subtle associations between delirium and long-term psychological outcomes, reflected by the absence of statistically significant differences in depression and trauma-related screening scores.

Another limitation is the absence of formal cognitive assessments in our study. While self-report questionnaires were distributed, cognitive impairment is optimally evaluated using standardized, observer-rated neurocognitive instruments such as the Mini-Mental State Examination (MMSE), the Montreal Cognitive Assessment (MoCA), or comprehensive neuropsychological test batteries, which were not available in the present retrospective design [28, 29].

Several predictors in the multivariable model (e.g., cerebrovascular or peripheral artery disease) showed extremely large regression coefficients and standard errors. This reflects quasi‑complete separation due to very small event counts in these categories, resulting in unstable and non‑interpretable effect estimates. These values must therefore be viewed as statistical artifacts rather than meaningful associations.

Furthermore, the study population was restricted to COVID-19 patients managed in a cardiothoracic ICU, a highly specialized setting which limits the generalizability of these findings to broader ICU cohorts. Consequently, these limitations advocate for (1) prospective, multicenter investigations (2) using standardized delirium assessment protocols.

## Conclusion

Our study demonstrates that delirium is a prevalent and multifaceted syndrome in critically ill COVID-19 patients treated in a cardiothoracic ICU, with important implications for clinical outcomes and resource utilization. Modifiable factors, particularly sedation and mobilization strategies, appear to play a central role in delirium risk and represent promising targets for prevention.

Given the known negative impact of delirium on recovery and healthcare resource utilization, prevention and management should be prioritized. Strategies should include standardized screening with validated tools such as CAM-ICU, optimization of sedation practices to minimize depth and duration, and early mobilization when feasible. By integrating early detection, protocolized sedation management, mobilization strategies, and structured post-ICU follow-up, it may be possible to reduce both the incidence and the long-term cognitive and psychological burden of delirium in this vulnerable patient population.

However, the nuances of delirium subtypes, study limitations, and long-term cognitive sequelae highlight the urgent need for prospective, multicenter research with standardized sedation and mobilization protocols to confirm our findings and to clarify causal mechanisms. Robust neuropsychological follow-up will be essential to improve delirium prevention, detection, and management in this complex patient population.

## Figures and Tables

**Fig. 1:**
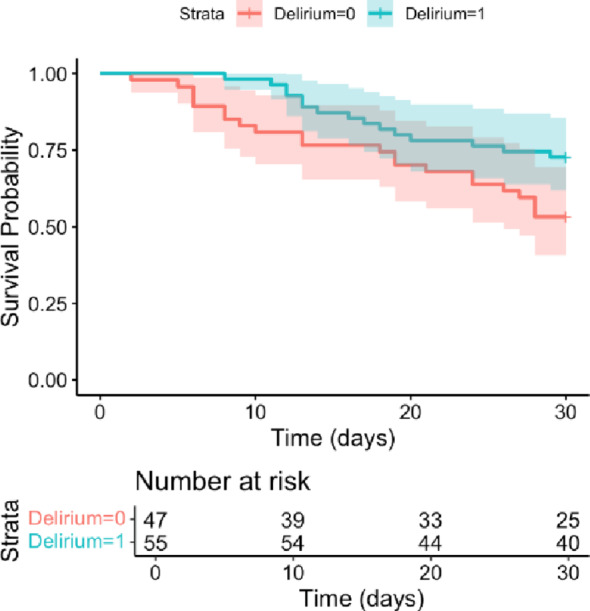
Kaplan-Meier survival curve for 30-day in-hospital survival (Delirium vs. no Delirium) *p* = 0,039

**Fig. 2:**
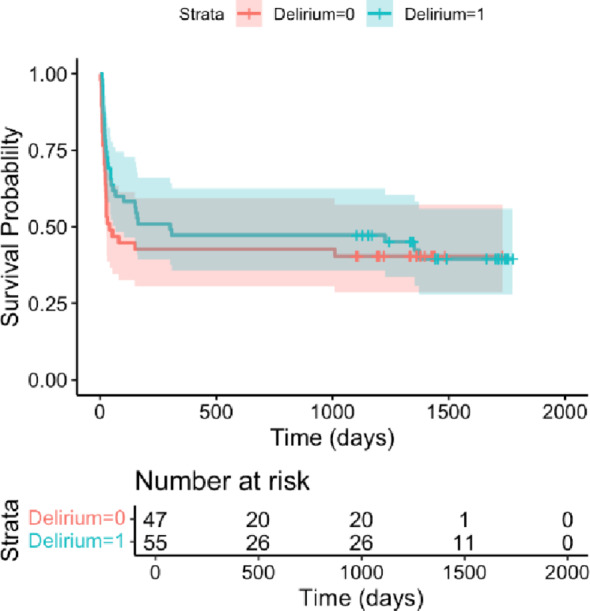
Kaplan-Meier survival curve for overall survival (Delirium vs. no Delirium) *p* = 0,400.

**Fig. 3:**
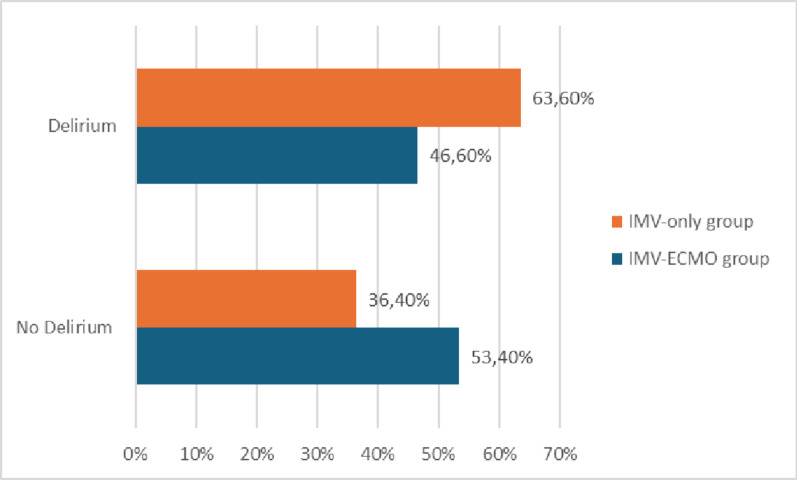
Incidence of delirium by treatment group (IMV‑only group vs. IMV–ECMO group), expressed as percentage of patients per group

## Data Availability

The datasets used and analysed during the current study are available from the corresponding author on reasonable request.

## Appendix

See Tables 1, 2 and 3; Figs. 1, 2 and 3.

**Table 1:** Baseline Characteristics and ICU Parameters by Delirium Status

	Delirium present	Delirium absent	*p*-value
Group size (*n*)	55	47	
Male sex	43	29	0,083
BMI^1^ (mean)	30,23	29,98	0,508
Age at admission (mean, in years)	59,38	55,68	0,136
Nicotine abuse	5	4	1,000
Diabetes	16	11	0,653
Hyperlipoproteinemia	13	6	0,205
Arterial hypertension	37	26	0,228
Coronary artery disease	23	11	0,060
Cerebrovascular disease	1	0	1,000
Peripheral artery disease	1	2	0,594
COPD^2^	5	6	0,750
Hemodialysis	22	19	1,000
Tracheotomy	32	31	0,540
Duration of invasive ventilation in days	32.82	19.87	0.436
Length of ICU stay in days (mean)	43.71	20.94	0.022*

* *p* < 0,05; considered statistically significant. ^1^BMI: Body Mass Index, ^2^COPD: chronic obstructive pulmonary disease, ICU: ^3^Intensive care unit

**Table 2:** Multivariate Logistic Regression: significant predictors of Delirium

Variable	Regression coefficient	SE (standard error)	Significance (*p*-value)	Hazard Ratio
ECMO only group = 1, IMV-only group = 2	1.315	0.565	0.020*	3.725
Sex; m = 1, f = 0	1.556	0.585	0.008*	4.740
BMI (mean)	0.052	0.038	0.178	1.053
Age at admission (in years)	0.013	0.018	0.484	1.013
Nikotine; yes = 1. no = 0	−0.397	0.861	0.645	0.672
Diabetes; yes = 1. no = 0	0.678	0.646	0.293	1.971
Hyperlipoproteinemia; yes = 1. no = 0	0.321	0.688	0.641	1.379
Arterial Hypertension; yes = 1. no = 0	0.520	0.547	0.341	1.683
Coronary artery disease yes = 1. no = 0	0.530	0.559	0.343	1.699
Cerebrovascular disease; yes = 1. no = 0	43.249	48823.991	0.999	0.000
Peripheral artery disease; yes = 1. no = 0	−24.066	27718.357	0.999	0.000
COPD; yes = 1. no = 0	0.450	0.812	0.579	1.568
Hemodialysis; yes = 1. no = 0	−0.614	0.519	0.237	0.541
Prone position; yes = 1. no = 0	−0.724	0.584	0.215	0.485
Duration of invasive ventilation in days	0.026	0.013	0.036*	1.027

*: *p* < 0,05 considered statistically signifikcant. ^1^BMI: Body Mass Index, ^2^COPD: chronic obstructive pulmonary disease

**Table 3:** Psychological Outcomes (BDI-II. ITQ. SF-12. ECR-R8) by Delirium Status

	Delirium present (mean)	Delirium present (median)	Delirium absent(mean)	delirium absent (median)	Significance Level (Mann-Whitney U Test)
BDI-II1	10.15	8.00	15.45	14.00	0.138
ITQ2	17.69	10.00	21.82	9.00	0.734
SF-12 PCS3	39.89	38.03	44.03	49.50	0.409
SF-12 MCS4	45.39	45.46	42.90	47.66	0.808
ECR5-R8 avoidance (single scores)	2.60	2.00	2.66	2.50	0.292
ECR-R8 avoidance (T-scores)	50.15	49.00	51.82	52.00	0.448
ECR-R8 anxiety (single scores)	2.04	2.00	2.05	1.25	0.424
ECR-R8 anxiety (T-scores)	51.69	51.00	49.54	46.00	0.191

^1^BDI-II: Beck Depression Inventory II, ^2^ITQ: International Trauma Questionnaire, ^3^SF-12 MCS: Short Form-12 Health Survey Physical Component Summary, ^4^SF-12 PCS: Short Form-12 Health Survey Mental Component Summary, ^5^ECR-R8: Experiences in Close Relationships – Revised

## Abbreviations

BD: Beck Depression Inventory
CAM-ICU: Confusion Assessment Method for the ICU
COPD: Chronic Obstructive Pulmonary Disease
DECAF: Delirium after Cardiac Surgery
ECMO: Extracorporeal Membrane Oxygenation
ICD: International Classification of Disease
ICU: Intensive Care Unit
IQR: Interquartile Range
ITQ: International Trauma Questionnaire
MCS: Mental Componant Summary
IMV: Invasive Mechanical ventilation
NMBA: Neuromuscular Blocking Agents
PCS: Physical Component Summary
PTSD: Post-traumatic Stress Disorder
RASS: Richmond Agitation Sedation Score
SD: Standard Deviation
SOFA: Sequential Organ Failure Assesment
VIF: Variance Inflation Factor
